# Supplementary material for: Plasmids Expressing shRNAs Specific to the Nucleocapsid Gene Inhibit the Replication of Porcine Deltacoronavirus In Vivo
Source: Animals (Basel). 2021 Apr 23;11(5):1216. doi: 10.3390/ani11051216 (PMC8145914; doi:10.3390/ani11051216)
Supplement: Supplementary file 1 [file animals-11-01216-s001.zip › Table S1.pdf]

Table S1. The value of villus height and crypt depth in small intestines of piglets under different treatments

	Group	Villus height (μm)		Crypt depth (μm)	
Duodenum	P+V	560.7	±125.6[A]	85.4	±49.2[A]
	P	1212.6	±134.5[B]	78.5	±41.5[AB]
	N+V	462	±154.2[C]	167.3	±61.6[C]
	N	995	±96.3[D]	180.4	±35.2[CD]
	C+V	197.5	±84.3[E]	176.5	±95.2[CE]
	C	1645.8	±354.6[F]	99.5	±48.2[AF]
Jejunum	P+V	576.2	±157.8[A]	68.2	±25.4[A]
	P	794.5	±145.6[B]	69.8	±44.1[AB]
	N+V	187.6	±102.3[C]	76.4	±33.5[AC]
	N	1056.2	±162.8[D]	89.6	±55.2[Ad]
	C+V	89.5	±42.1[E]	95.4	±43.2[E]
	C	1432.9	±268.4[F]	102.6	±65.2[F]
Ileum	P+V	653.2	±243.1[A]	67.5	±44.4[A]
	P	1128.53	±257.5[B]	98.5	±66.3[B]
	N+V	342.2	±250.4[C]	153.2	±78.4[C]
	N	1254	±365.4[D]	97.5	±26.2[BD]
	C+V	214.5	±78.6[E]	166.7	±48.6[CE]
	C	1680.8	±543.2[F]	121.6	±54.3[F]

Note: letter indicates that the difference is not significant ($P>0.05$). The same uppercase letters and different lowercase letters indicate significant difference ($0.05>P>0.01$). Different lowercase letters and different uppercase letters indicate that the difference is statistically highly significant ($P<0.01$).
