# Supplementary figures and images for: Plasmids Expressing shRNAs Specific to the Nucleocapsid Gene Inhibit the Replication of Porcine Deltacoronavirus In Vivo
Source: Animals (Basel). 2021 Apr 23;11(5):1216. doi: 10.3390/ani11051216 (PMC8145914; doi:10.3390/ani11051216)

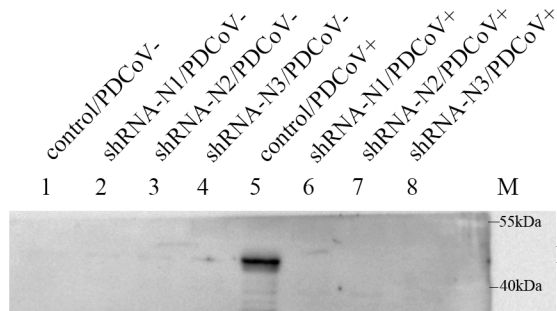

PDCoV N

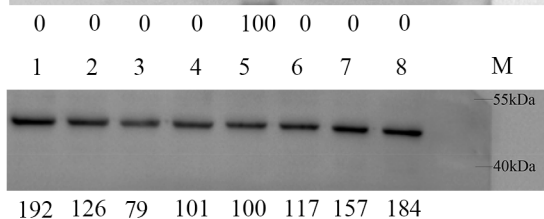

$\beta$ -actin

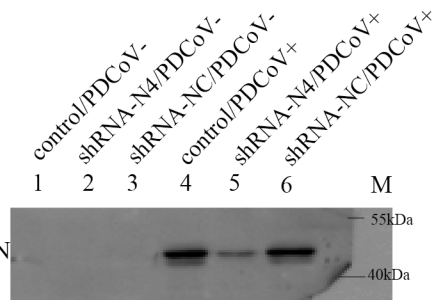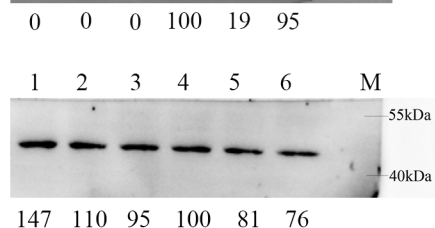

Supplement: Supplementary file 1 [file animals-11-01216-s001.zip › Figure 2 corrected.pdf]

control/PDCoV-  
 Double-shRNA-N1/PDCoV-  
 control/PDCoV+  
 Double-shRNA-N1/PDCoV+

1 2 3 4 M

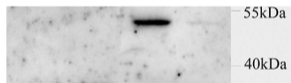

PDCoV N

0 0 100 0

M 1 2 3 4

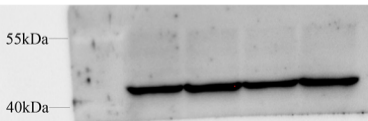

$\beta$ -actin

113 144 100 147

Supplement: Supplementary file 1 [file animals-11-01216-s001.zip › Figure 5b.pdf]

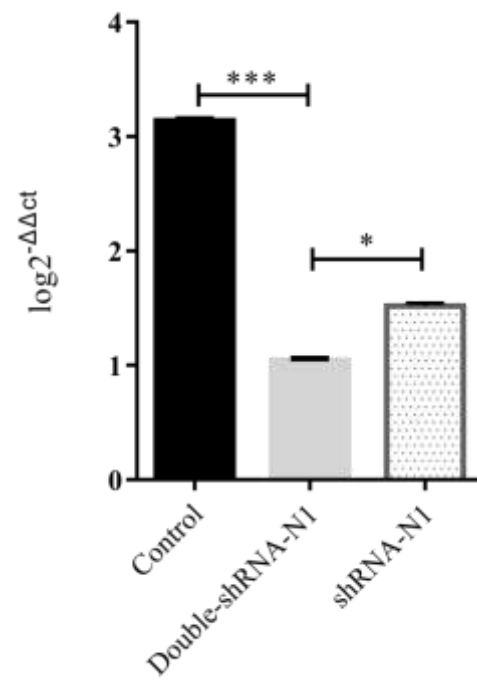

Figure S1: The reduction of PDCoV RNA by double-shRNA-N1 or single shRNA-N1 expressing plasmids

Supplement: Supplementary file 1 [file animals-11-01216-s001.zip › Figure S1.pdf]
